# Ewing sarcoma and paediatric cancer burden: we need more robust registries

**DOI:** 10.1016/j.lana.2026.101683

**Published:** 2026-10-09

**Authors:** The Lancet Regional Health – Americas

Since 2007, at least nine unrelated children from Ladera Ranch, a small community in Orange County, Southern CA, USA, have been diagnosed with Ewing sarcoma, a very rare tumour of bone and surrounding soft tissues that mainly affects children and young adults. If the tumour has not spread, the 5-year survival is nearly 75%. The report of a substantial number of Ewing sarcoma cases within a small area, considering the rarity of the disease (an annual incidence of nearly three cases per million people in the USA), has left community members, patients and families, and experts with many unanswered questions. Concerns have been raised that possible environmental factors may be involved. However, as it is the case for most childhood cancers, Ewing sarcoma does not yet have an established cause.

The report in Ladera Ranch appears to be the largest series of Ewing sarcoma cases limited to a small area. Clusters of other types of cancer were described elsewhere in the USA between the 1960s and the 1980s. For instance, unusually high occurrences of leukaemia and neurological cancer were reported in Tom's River, NJ. Contaminated drinking water and air pollution were identified as the potential causes. Clusters of leukaemia were also reported in a city in Woburn, MA, where contaminated drinking water was also indicated as the associated factor. These examples of clusters of cancer cases suggest that a potential environmental factor should not be ruled out as an explanation for the cases reported in Ladera Ranch. Ewing sarcoma is not believed to be heritable, which suggests that epigenetics could play a role in its pathogenesis. Whether exposure to environmental chemicals present in water, food, soil (e.g., in parks) or air could have triggered mutations in the genes involved in Ewing sarcoma (EWSR1 and FLI1) remains to be established.

In the USA, Ewing sarcoma accounts for only 1% of all paediatric cancers, whereas leukaemia, CNS cancers and lymphomas are the most common paediatric cancers, comprising nearly two thirds of all cancers affecting children and adolescents. While cancer in adults represent most cancer cases (about 95.5%), globally, 377,000 incident childhood cancer cases and 144,000 deaths were estimated in 2023. The Americas and South-East Asia had the lowest percentage of global childhood cancer cases (13.1%), whereas the African and Western Pacific regions had the highest percentages (24.4% and 24.5%, respectively). The African region had by far the highest number of deaths (34.1%).

In this issue, a study by Sanches and colleagues shows that lymphoid leukaemia mortality is increasing in states from the north and northeast regions of Brazil, while rates are stable or decreasing in southern and southeastern states. Age-specific mortality rates were higher in states with lower socioeconomic settings. Another study by Cerda and colleagues describes the National Childhood Cancer Registry in Chile that is used to track incidence, mortality, survival, and the distribution of diagnoses in children aged 0–14 years. Their study shows that cancer incidence increased from 137.4 per million in 2007 to 143.0 per million in 2023. Similar to global patterns, leukaemia, central nervous system cancer and lymphoma are also the most common types of childhood cancer in Chile. Global Burden of Disease data report that the age-standardised incidence of childhood cancer in Latin America and the Caribbean increased by 18.5% between 1990 and 2023, although there is considerable uncertainty in the estimates.

Addressing childhood cancer burden requires strong health systems including comprehensive data registries. Unfortunately, data collection in cancer is still not robust across the region. In Latin America, high-quality population-based national cancer registries are available only in Costa Rica and Uruguay. The cluster of Ewing sarcoma cases in southern California remind us of the need for research on the aetiology of Ewing sarcoma and other cancers affecting people early in their lives. Cancer registries and surveillance are critical for understanding disease patterns, for effective policy implementation, and for hypothesis-generating studies. Establishing high-quality national cancer registries in the region is critical. The Pan American Health Organization's launch of the Regional Childhood Cancer Observatory on Sept 25, 2026, is a timely step towards strengthening regional monitoring and collaboration. Stronger multidisciplinary collaborations between molecular biologists, geneticists, data scientists, toxicologists, public health specialists, epidemiologists, and health-care providers are needed to accelerate progress in childhood cancer prevention, diagnosis, and treatment. Funding, adequate infrastructure, accurate reporting, and linkage between clinical and mortality data are essential to build robust, high-quality nationwide cancer registries.

